# The invasive shrub *Prosopis juliflora* enhances the malaria parasite transmission capacity of *Anopheles* mosquitoes: a habitat manipulation experiment

**DOI:** 10.1186/s12936-017-1878-9

**Published:** 2017-07-05

**Authors:** Gunter C. Muller, Amy Junnila, Mohamad M. Traore, Sekou F. Traore, Seydou Doumbia, Fatoumata Sissoko, Seydou M. Dembele, Yosef Schlein, Kristopher L. Arheart, Edita E. Revay, Vasiliy D. Kravchenko, Arne Witt, John C. Beier

**Affiliations:** 1 Department of Microbiology and Molecular Genetics, IMRIC, Kuvin Centre for the Study of Infectious and Tropical Diseases, Faculty of Medicine, Hebrew University, Jerusalem, Israel; 2 Malaria Research and Training Center, Faculty of Medicine, Pharmacy and Odonto-Stomatology, University of Bamako, Bamako, BP 1805 Mali; 3 Department of Public Health Sciences, Miller School of Medicine, University of Miami, Miami, FL USA; 4 Department of Anatomy and Cell Biology, Bruce Rappaport Faculty of Medicine, Technion, 34995 Haifa, Israel; 5 Department of Zoology, Tel Aviv University, Tel Aviv, Israel; 6 CABI Africa, Box 633-00621, Nairobi, Kenya

**Keywords:** Invasive plants, *Anopheles gambiae* complex, *Prosopis juliflora*, Mali

## Abstract

**Background:**

A neglected aspect of alien invasive plant species is their influence on mosquito vector ecology and malaria transmission. Invasive plants that are highly attractive to *Anopheles* mosquitoes provide them with sugar that is critical to their survival. The effect on *Anopheles* mosquito populations was examined through a habitat manipulation experiment that removed the flowering branches of highly attractive *Prosopis juliflora* from selected villages in Mali, West Africa.

**Methods:**

Nine villages in the Bandiagara district of Mali were selected, six with flowering *Prosopis juliflora*, and three without. CDC-UV light traps were used to monitor their *Anopheles* spp. vector populations, and recorded their species composition, population size, age structure, and sugar feeding status. After 8 days, all of the flowering branches were removed from three villages and trap catches were analysed again.

**Results:**

Villages where flowering branches of the invasive shrub *Prosopis juliflora* were removed experienced a threefold drop in the older more dangerous *Anopheles* females. Population density dropped by 69.4% and the species composition shifted from being a mix of three species of the *Anopheles gambiae* complex to one dominated by *Anopheles coluzzii*. The proportion of sugar fed females dropped from 73 to 15% and males from 77 to 10%.

**Conclusions:**

This study demonstrates how an invasive plant shrub promotes the malaria parasite transmission capacity of African malaria vector mosquitoes. Proper management of invasive plants could potentially reduce mosquito populations and malaria transmission.

## Background

Blood feeding of female mosquitoes is mainly utilized for egg development, but repeated meals also allow the transmission of various pathogens such as malaria parasites. Energy for all other life-sustaining activities of both females and males is provided by plant sugars, usually nectar from flowers [1, 2]. Considering the importance of sugar in mosquito biology, it is surprising that there is a dearth of information on where they obtain sugars in nature. Particularly obscure is the role invasive alien plants play in the survival and vectorial capacity of mosquitoes. Some invasive plants that are abundant and widespread on the African continent are attractive to *Anopheles* species and can be used as sources of sugar meals [3, 4]. Because they are more widespread and abundant, and actively grow and flower for longer periods than related native species, invasive plants could significantly contribute to mosquito longevity, and thereby enhance malaria transmission potential [5]. Increased survival of the vector, even by a day or two, can greatly increase the number of mosquitoes that live to become infectious. This is because it is estimated that in high transmission areas, it takes at least 12 days for malaria parasites to undergo sporogonic development before migrating to the salivary glands of vector mosquitoes [6, 7], yet only about 10% of these same mosquitoes live 12 days [7].

*Prosopis juliflora* (Fabaceae; mesquite) is native to Central and South America [6] and was introduced to new environments across the world in the late 1970s to early 80s in an attempt to reverse deforestation and desertification [8, 9]. In its introduced range, *Prosopis juliflora* is utilized for construction materials, fuel wood, charcoal, and fodder [8]. However, it grows rapidly, produces copious amounts of seeds and is tolerant of a wide range of climatic regimes and soil types [10], which have contributed to making *Prosopis juliflora* one of the worst invasive alien plants in many parts of the world [9, 11]. This spiny shrub, or small tree, now occupies millions of hectares in Mali, Chad, Niger, Ethiopia, Sudan, Kenya, Tanzania and elsewhere, eroding the natural resource base on which millions of people depend, and in some instances, even driving conflict as people compete for access to grazing land and water [11, 12].

**Fig. 1:**
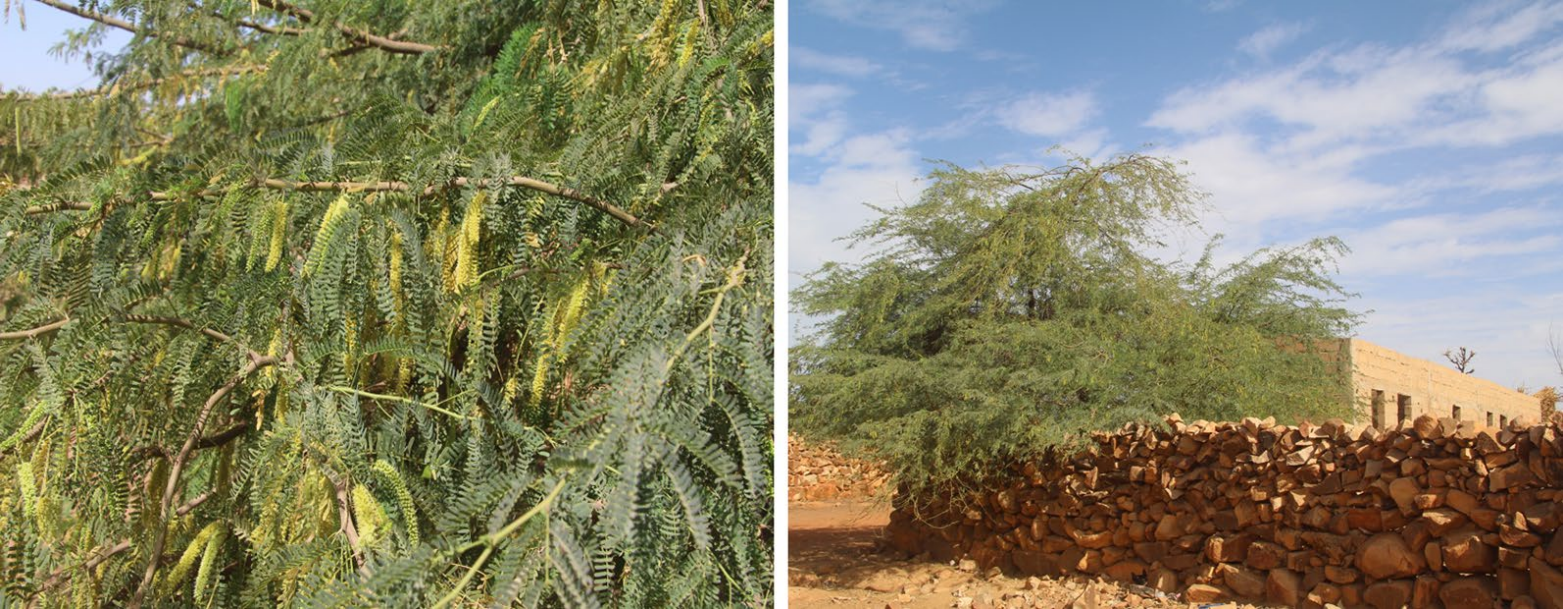
*Prosopis juliflora* with inflorescences (*Left*). *Prosopis juliflora* in a village in the Bandigara District, Mali (*Right*)

The objective of this study was to define the importance of an invasive plant as a resource for mosquito survival, and a contributing factor to continuing transmission of malaria, especially during dry periods when sugar sources from native plants are largely unavailable. In preparation for this trial, the local plant species most attractive to mosquitoes, that had been identified in a previous survey [13], were compared with the alien species *Prosopis juliflora*, *Acacia salicina* (Fabaceae; willow wattle) and *Eucalyptus cladocalyx* (Myrtaceae; sugar gum) and it was found that their high attraction amounted to indices of 24, 11 and 7, respectively (unpublished). In this study, in villages in Mali during the dry season, the following parameters were assessed: the role of flowering *Prosopis juliflora* on the density of mosquito populations, their species composition, their sugar feeding status and age structure. Nine villages in the Bandiagara District were selected, six with flowering *Prosopis juliflora*, and three without, and then vector populations were monitored (Fig. 1). Afterwards, the *Prosopis juliflora* flowering branches were removed from three of the six villages where the invasive shrub was actively growing, mosquito populations continued to be monitored. The hypothesis was that the villages with flowering *Prosopis juliflora* would harbor larger and older mosquito populations than the sugar-poor environment found in villages that are devoid of *Prosopis juliflora*. The rationale was that if the invasive plants are an important source of sugar during the dry season, then they would contribute to malaria vector survival effectively extending the transmission season.

## Methods

### Study sites

The study was conducted from mid-January to early February 2016 in nine villages along the Dogon plateau, in the Bandiagara District of central Mali. The study villages were located near the margins of the inland delta of the Niger River beginning about 650 km northeast of Bamako. Along the roads connecting the villages were clusters of three to five ponds for collecting rainwater, varying in size from 3000 to 10,000 m^2^, that were created artificially to be used as a water supply for livestock and as rice paddies in the shallower areas. These ponds are surrounded by arid vegetation and larval surveys revealed high densities of *Anopheles gambiae* s.l. Villages were between 5 and 8 km from each other, and around 5 km away from any other area where mosquitoes breed in large numbers. The areas around the villages were dominated by grassland. The region is semi-arid with the rainy season between July and September with peak malaria transmission occurring in October [14]. Prevalence of *Plasmodium falciparum* infection ranges from 45% during the dry season, to more than 65% at the end of the rainy season [14].

### Study design and mosquito trapping

The study was conducted over a total of 18 days. There were 8 days of pre-intervention monitoring from 10 to 18 January 2016, followed by the removal (cutting) of flowering branches in three selected villages (19–20 January). Post-intervention monitoring was from 1 to 8 February. The study was conducted during the dry season, when plants other than *Prosopis juliflora*, which may act as sugar sources for mosquitoes, were not flowering.

Monitoring of the mosquito populations was done using eight CDC-UV light traps per village (model 1212, John W. Hock, Gainesville, FL, USA), without bait, suspended from buildings, approximately 1.5–2.0 m above the ground. Traps were hung outdoors, at dusk, in fixed locations, and were spread randomly throughout villages, at least 20 m apart. Trap catches were collected 1 h after sunrise. Mosquitoes were either frozen, stored in 70% ethanol or processed right away.

### Assessing population size and species composition

*Anopheles gambiae* s.l. mosquitoes caught in the CDC traps were counted to determine overall population size in each village. Sub-samples of catches (n = 90) from each village were stored in 70% ethanol for morphological species identification [15]. A random subsample of 25 mosquitoes per village were identified by PCR [16].

### Age grading of female mosquitoes

Pre- and post-intervention samples of 100 female mosquitoes from each village were analysed and the physiological age of random samples of female mosquitoes was determined by examination of ovaries, which were removed from the mosquito and dissected in a drop of PBS under a dissecting microscope, to expose and count the dilatations in ovarioles [17].

### Sugar feeding status

Gut sugar content was determined by a modified cold anthrone test for fructose [18]. Crushed mosquitoes were examined after incubation and meal size was estimated subjectively based on the degree of blue–green colour [19].

### Statistical analysis

A generalized linear mixed model was used for the population size and gender composition data. Separate analyses were conducted for counts of females and males. The data showed marked over-dispersion, therefore, a negative binomial regression model was used. The fixed effects were groups (groups of the three villages being monitored) and time (pre- and post-intervention) with an interaction of group and time. A random intercept was included with villages nested within groups as the error term. An unstructured covariance matrix was used to represent the correlated data structure. Planned comparisons were made between times within each group and among groups within each time.

A generalized linear model was used for the female age data: a logistic regression model was used. The fixed effects were groups (groups of villages being monitored) and time (pre- and post-intervention) with an interaction of group and time. Planned comparisons were made between times within each group and among groups within each time.

A generalized linear model was used for the sugar feeding data. Separate models were run for males and females. A logistic regression model was used. The fixed effects were groups (groups of villages being monitored) and time (pre- and post-intervention) with an interaction of group and time. Planned comparisons were made between times within each group and among groups within each time.

In addition, we used a generalized linear model for the species composition of populations. The fixed effects were groups (groups of villages being monitored), time (pre- and post-intervention), and species (*Anopheles arabiensis*, *Anopheles coluzzii*, and *An. gambiae*) with interactions of group and time, group and species, and time and species. Planned comparisons were made between times within each species, among species at each time, among species within each group, and among groups within each species. SAS 9.3 (SAS Institute, Inc.; Cary, NC) was used for all analyses. The 0.05 alpha level was used to determine statistical significance.

## Results

### Population size

In the three negative control villages, those without *Prosopis juliflora*, the average (±SE) catch per trap in the pre-intervention monitoring period was 3.58 ± 0.32 females and 0.56 ± 0.11 males, similar to what was found at the end of the study (3.31 ± 0.43 females and 0.70 ± 0.09 males).

In the three positive controls, that is villages with *Prosopis juliflora* where the inflorescences were not removed, the average (±SE) catch per trap was 6.52 ± 0.60 females and 3.10 ± 0.37 males at the initiation of the trial. Similar catches were recorded at the end of the trial (6.79 ± 0.58 females and 2.6 ± 0.23 males).

In the three experimental villages with *Prosopis juliflora*, where intervention occurred by removing the flowering branches, the initial average (±SE) catch was 11.00 ± 0.93 females and 6.00 ± 0.66 males, dropping to 4.5 ± 0.67 females and 0.7 ± 0.16 males, post-intervention (after the inflorescences had been removed).

**Fig. 2:**
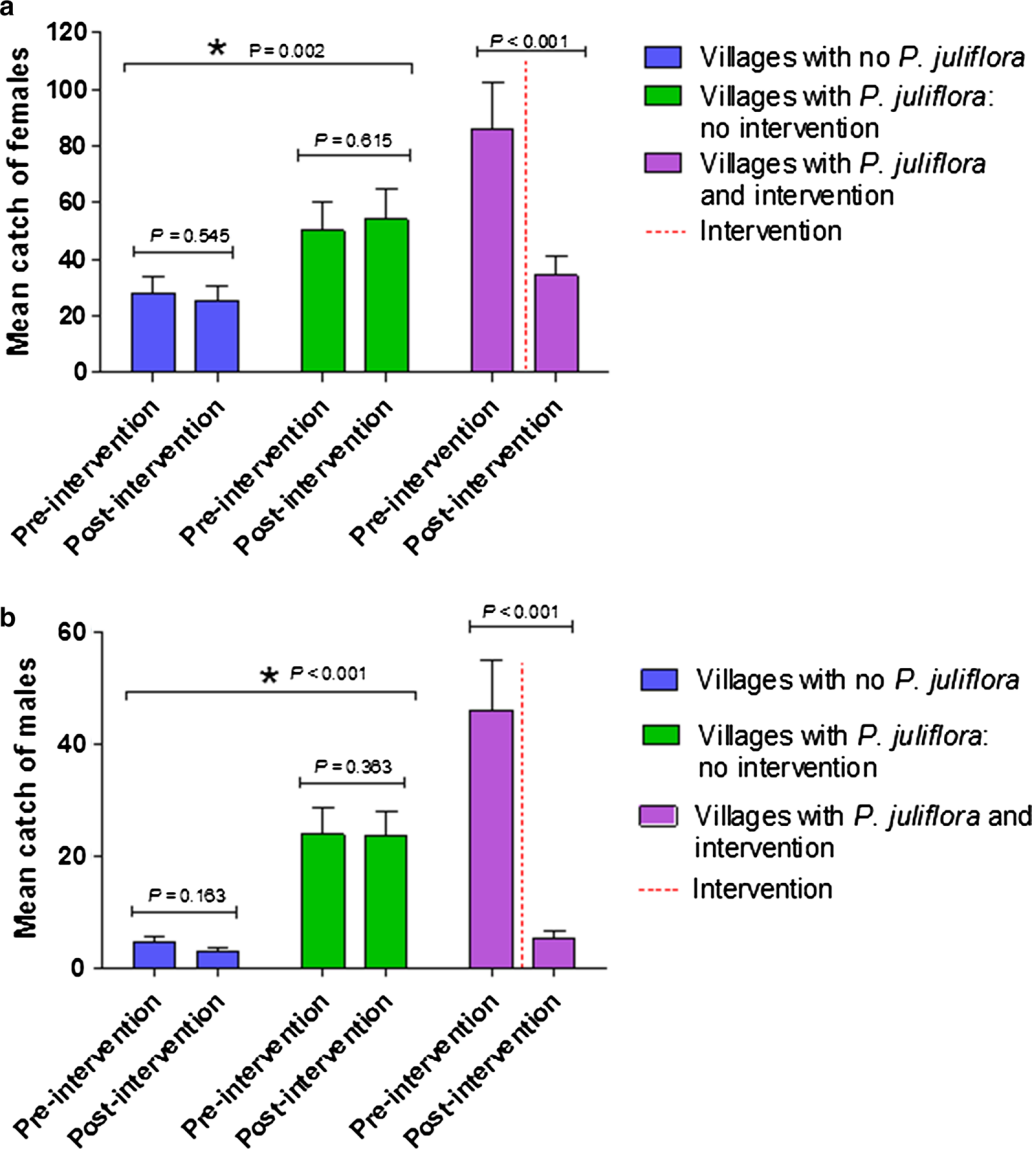
Mean catches of female (**a**) and male (**b**) mosquitoes in the pre- and post-intervention monitoring periods. *Asterisk* represents significant differences between village groups

Total trap catches for each three-village group were pooled, and the mean population sizes among each village group of females and males were compared (Fig. 2a, b). Villages without *Prosopis juliflora* had distinctly smaller mosquito populations than villages with *Prosopis juliflora*, and after removal of the flowering branches from *Prosopis juliflora* in the selected villages, the total population of female and male *Anopheles* spp. decreased significantly. The mean number of females decreased by 59.9% and the mean number of males decreased by 88.5% (*P* < 0.05).

### Female age grading

**Fig. 3:**
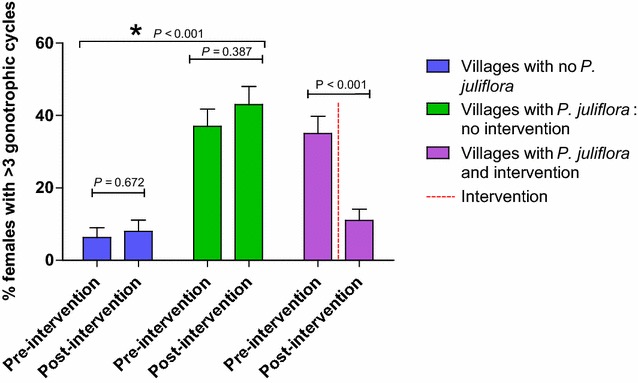
Age structure of *Anopheles* spp. female populations in villages with and without *Prosopis juliflora* in the pre- and post-intervention monitoring periods. *Asterisk* represents significant differences between village groups

In villages with no *Prosopis juliflora*, a low proportion of old females, 6.25% in the pre-intervention monitoring period, and 8% in the post-intervention monitoring period, had undergone three or more gonotrophic cycles (Fig. 3). In villages with *Prosopis juliflora*, where flowering branches were not removed, the proportion of older females was much higher (37% in the pre-intervention and 43% in the post-intervention monitoring period). Prior to the removal of inflorescences, a comparable 35% of females had undergone three or more gonotrophic cycles, dropping down to a statistically significant level of 11% after removal of flowering branches (Fig. 3; *P* < 0.05), which is similar to that found in villages (8%) with no *Prosopis juliflora*.

### Sugar feeding status

**Fig. 4:**
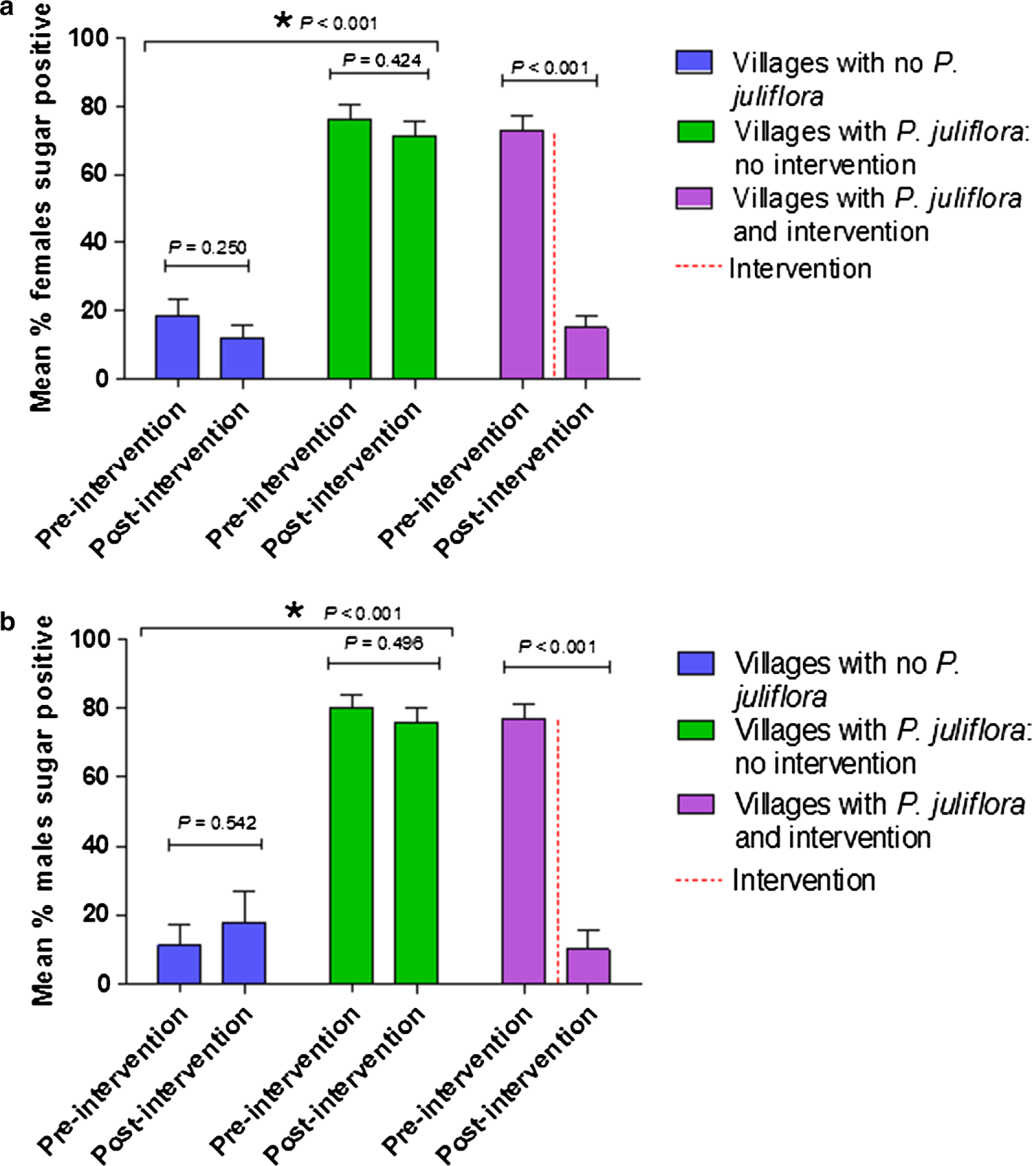
Sugar feeding status of **a** female and **b** male *Anopheles gambiae* in villages with and without *Prosopis juliflora* in the pre- and post-intervention monitoring periods. *Asterisk* represents significant differences between village groups

Anthrone testing showed that villages with flowering *Prosopis juliflora* have a significantly higher proportion of sugar-fed female and male mosquitoes than villages without this invasive shrub. When the flowering branches are removed, the effect on the proportion of sugar positive mosquitoes is dramatic. In these villages, there was a fivefold decrease in the mean number of sugar positive females and a nearly eightfold decrease in sugar positive males (Fig. 4a, b). In villages where *Prosopis juliflora* flowers were removed, the initially large sugar meals were replaced by small ones.

### Species composition

**Fig. 5:**
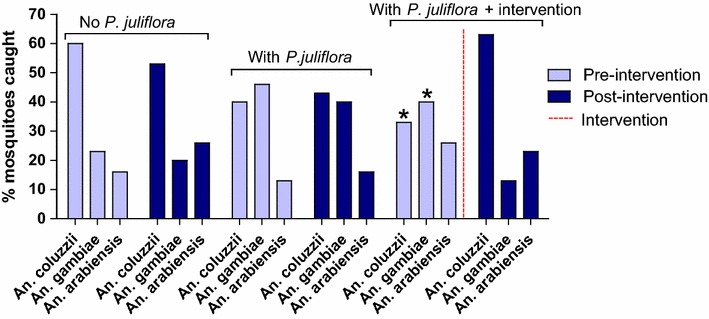
Species composition in villages with and without *Prosopis juliflora* in the pre- and post-intervention monitoring periods. *Red dashed line* indicates inflorescence removal (intervention). *Asterisks* represent significant differences between pre- and post-intervention population density; *P* = 0.041 for the difference in *Anopheles coluzzii* population density and *P* = 0.045 for the *Anopheles gambiae* s.s. population

In villages without *Prosopis juliflora*, and those where *Prosopis juliflora* was present, but where the inflorescences were not removed, the relative abundance of various mosquito species remained stable during the pre- and post-intervention monitoring periods (Fig. 5). In contrast, there were obvious changes in the species composition in villages where branches with flowers were removed. Post-removal, the proportion of *An. coluzzii* in the diminished population increased by 50% and there was a marked drop of 27% in the relative number of *An. gambiae* s.s.

## Discussion

Previous studies have demonstrated that mosquitoes in arid areas feed on rare floral nectar of local plant species [20–22], but here it is shown what happens to mosquito population dynamics when there is an abundant source of sugar. This is the situation that occurs in the presence of the inflorescences of the widespread invasive alien species, *Prosopis juliflora*. In this study, experimental habitat manipulation data from the field is presented that demonstrates the beneficial effect of *Prosopis juliflora* on vector mosquito populations. The wide distribution and density of these plants and how they affect malaria transmission in Mali is considered, particularly during the dry season when scarcity of other sugar sources in the local flora limits mosquito longevity. The presence or absence of *Prosopis juliflora* in villages has a significant influence on the size of the mosquito population in general, on their species composition, on the sugar feeding status and, the age structure of female populations.

Villages invaded by *Prosopis juliflora* have higher populations of *An. gambiae* s.l. than those where this plant is absent. Mean CDC trap catches of *An. gambiae* s.l. females in villages where flowering *Prosopis juliflora* was present was about twice as high as that in villages without flowering *Prosopis juliflora*. The effect was even more pronounced in males where the population was five to eight times higher in villages with *Prosopis juliflora* than in those without. When the flowering branches, evidently an abundant dry season sugar source, were removed from villages with *Prosopis juliflora*, the population of males and females dropped almost ninefold and more than twofold, respectively.

This is in agreement with a previous study [23], in southern Israel, that found that *Anopheles sergentii* in a sugar-poor oasis, i.e. in the absence of *Acacia tortilis* and *Acacia radianna* (Fabaceae) trees, had smaller population sizes (37,494) than in those where this native plant species was abundant (85,595). This is further supported by studies which have indicated that a lack of sugar results in reduced male *An. gambiae* survival and mating capability contributing to lower insemination rates in females [28] which may contribute to reduced population levels. This study also found that *An. sergentii* had lower survival rates (0.72 vs. 0.93), and prolonged gonotrophic cycles (3.33 vs. 2.36 days). Most telling was the fact that vectorial capacity was more than 250-fold higher in sugar-rich oasis than that in sugar-poor sites.

Older female mosquitoes that feed often on human hosts have an increased vectorial potential. The female *Anopheles* in villages with *Prosopis juliflora* were older since many of them had passed three or more gonotrophic cycles and they were thus more effective dangerous vectors. Villages with the invasive plant contained around six times more of these older females than those without and there was a threefold decrease in the proportion of this group as soon as the flowering branches were removed.

In the current study, there was a marked difference in the proportion of sugar positive male and female mosquitoes depending on whether *Prosopis juliflora* was present or absent. Previous studies found that *An. gambiae* s.s. females lived longer when sugar-fed [24, 25] with survivorship greatly enhanced with access to sugar-rich plant species. It is, therefore, not surprising that the presence or absence of a rich sugar source like *Prosopis juliflora* had a significant effect on mosquito populations with four to six times more females, and up to eight times more males, than in villages without this invasive shrub. Conclusive evidence for the role of *Prosopis juliflora* is that the removal of inflorescences resulted in a drastic eight- and fivefold decrease in sugar-fed males and females respectively.

Species composition, while remaining consistent during the pre- and post-intervention monitoring in villages where there were no interventions, changed markedly in villages where flowering branches were removed. In the latter villages, the number of *An. coluzzii*, roughly doubled while the number of *An. gambiae* s.s. fell by nearly a third. In fact, the relative proportions of mosquito species after removal of *Prosopis juliflora* blossoms came to more closely resemble that in villages where this plant was wholly absent. It, therefore, shows that the removal of flowering branches removed the major sugar source of *An. gambiae* s.s. making way for a higher proportion of *An. coluzzii*. The malaria vector *An. gambiae* s.s. seems to be highly dependent on the blossoms of *Prosopis juliflora,* whereas *An. coluzzii* is more adapted to arid habitats [26] and can thrive even in the absence of the inflorescences.

Current strategies for malaria vector control used in most African countries are not sufficient to achieve successful malaria control [27]. Vector control in Africa needs to be improved because even very low levels of malaria parasite transmission confound efforts to reduce malaria prevalence [28].

This study helps understanding of how aspects of mosquito biology, other than blood feeding, influence the vectorial capacity of mosquitoes. Control of invasive plants could be a new way to change inherently high transmission areas to low transmission areas, making elimination by combinations of vector control methods more feasible. If biocontrol or community efforts to manage invasive plants are developed and implemented, they can provide an environmentally reasonable sustainable strategy in reducing the incidence of malaria. Improved management of invasive plants will also provide a host of other benefits, including increased incomes for local communities.

Supporting malaria vectors with a source of energy in the form of sugar is not the only disadvantage of the spread of *Prosopis juliflora.* These plants, among others, encroach on paths, homes and other structures in villages, invade crop- and pasturelands and their thorns are known to cause injuries [9, 29]. *Prosopis juliflora* invasions have also contributed to the abandonment of agricultural land, and in some cases also of homes and small villages [9, 29]. These negative impacts, and the results of our study, clearly demonstrate that the control of *Prosopis juliflora* may not only reduce the incidence of malaria but may benefit a range of other sectors, and in so doing, will contribute to a better quality of life for many communities in large parts of Africa. The introduction of host specific and damaging biological control agents, such as *Evippe* sp., may be a cost-effective and safe way of reducing the impact of invasive *Prosopis* species in Africa and elsewhere [30].

## Conclusions

Future studies might concentrate on many invasive plant species that are problematic in Africa and can be attractive to vector populations. It may be worthwhile to abstain from the introduction of exotic plants that have the potential to become invasive, not only because of their potential negative impacts on the environment and livelihoods, but because some of them may have negative significant consequences for public health and specifically for malaria.
